# Supplementary material for: Distinct neural bases of subcomponents of the attentional blink
Source: eLife. 2025 Jun 24;13:RP97098. doi: 10.7554/eLife.97098 (PMC12187135; doi:10.7554/eLife.97098)
Supplement: Supplementary file 1. [file elife-97098-supp1.docx]

Supplementary Information for

Distinct neural bases of subcomponents of the attentional blink

Swagata Halder^1^*, Deepak Velgapuni Raya^1^, Devarajan Sridharan^1,2^*

^1^Centre for Neuroscience, Indian Institute of Science, Bangalore 560012, India, and

^2^Computer Science and Automation, Indian Institute of Science, Bangalore 560012, India

Supplementary file 1

Supplementary tables.

**Table A. Attentional blink effect on psychometric measures.**

2-way ANOVA analysis with the psychometric measures as dependent variables and lags and contrasts as independent factors.

| **Psychometric measures** | **Analysis of Variance (ANOVA)** | | |
| --- | --- | --- | --- |
|  | Main effect of lag  (F, p, BF) | Main effect of contrast  (F, p, BF) | Interaction effect  (F, p, BF) |
| Hit | **55.78, <0.001, >10^3^** | **35.52, <0.001, >10^2^** | 2.23, 0.149 (n.s.), 0.538 |
| Miss | **10.96, 0.003, 20** | **21.6, <0.001, >10^2^** | 2.31, 0.142 (n.s), 0.597 |
| Misidentification | **42.45, <0.001, >10^3^** | 3.18, 0.090 (n.s.), 0.367 | 0.02, 0.889 (n.s), 0.276 |
| FA | **6.9, 0.015, >10^3^** | N/A | N/A |
| CR | **6.9, 0.015, >10^3^** | N/A | N/A |

**Table B. Partial correlations of ERP amplitudes with detection and discrimination sensitivity.**

Magnitude of blink induced deficit in ERP amplitudes, and partial correlation between ERP amplitudes, and detection (discrimination) d’, while controlling for the confounding effect of discrimination (detection) d’ (Methods). **Bold** entries correspond to significant partial correlations.

| **ERP components** | **Blink deficit (mean ± s.e.m)** | **Partial correlation**  **with sensitivity (d’)** | |
| --- | --- | --- | --- |
|  |  | Partial correlation with detection d’ | Partial correlation with discrimination d’ |
| P1 (parietal) | 0.25 ± 0.16,  p = 0.231 | r_p_ = -0.06  p = 0.935 | r_p_ = -0.11  p = 0.816 |
| P2 (frontocentral) | 0.19 ± 0.07,  p = 0.021 | r_p_ = 0.05  p = 0.999 | r_p_ = 0.23  p = 0.120 |
| N2p (occipitoparietal) | **-0.47 ± 0.12,**  **p = 0.003** | **r_p_ = -0.34**  **p < 0.001** | r_p_ = -0.01  p = 0.970 |
| P3 (parietal) | **0.45 ± 0.09,**  **p < 0.001** | **r_p_ = 0.30**  **p < 0.001** | r_p_ = 0.14  p = 0.748 |

**Table C. Partial correlation of fronto-parietal coherence with detection and discrimination sensitivity.**

Partial correlation between left and right frontoparietal beta coherence and detection and discrimination sensitivity. Other conventions are the same as in Table B.

| **Hemisphere** | **Beta band** | **Partial correlation**  **with sensitivity (d’)** | |
| --- | --- | --- | --- |
|  |  | Partial correlation with detection d’ | Partial correlation with discrimination d’ |
| Left fronto-parietal | High beta  (20-30 Hz) | r_p_ = -0.05  p = 0.316 | **r_p_ = 0.22**  **p = 0.018** |
|  | Low beta  (13-19 Hz) | r_p_ = 0.05  p = 0.728 | r_p_ = -0.03  p = 0.728 |
| Right fronto-parietal | High beta  (20-30 Hz) | r_p_ = -0.01  p = 0.470 | r_p_ = 0.21  p = 0.1 |
|  | Low beta  (13-19 Hz) | r_p_ = -0.04  p = 0.680 | r_p_ = 0.05  p = 0.682 |

**Table D. Partial correlation between neural measures and neural distances in the detection and discrimination dimensions.**

Partial correlation between neural measures (N2P and P3 ERP amplitudes and left frontoparietal high-beta coherence) and neural distances in detection (discrimination) dimension while controlling for the confounding effect of the neural distance in the discrimination (detection) dimension.

| **Neural measure** | **Partial correlation with**  **neural distance (ɳ)** | |
| --- | --- | --- |
|  | Neural distance in the detection dimension (\|\|**ɳ_det_**\|\|) | Neural distance in the discrimination dimension (\|\|**ɳ_dis_**\|\|) |
| N2p amplitude | **r_p_ = -0.31, p = 0.02** | r_p_ = 0.07, p = 0.330 |
| P3 amplitude | **r_p_ = 0.46, p < 0.001** | r_p_ = 0.15, p = 0.160 |
| Left fronto-parietal high- beta (20-30 Hz) coherence | r_p_ = 0.07, p = 0.255 | **r_p_ = 0.26, p = 0.039** |
